# "Drop in" gastroscopy outpatient clinic - experience after 9 months

**DOI:** 10.1186/1471-230X-12-12

**Published:** 2012-02-01

**Authors:** Gert Huppertz-Hauss, Lubomir Chengarov, Stein Dahler, Anita Jørgensen, Volker Moritz, Jørn Paulsen, Geir Hoff

**Affiliations:** 1 Department of Gastroenterology, Medical Clinic, Telemark Hospital, 3710 Skien, Norway

**Keywords:** endoscopy, gastroscopy, outpatient clinic, waiting lists

## Abstract

**Background:**

Logistics handling referrals for gastroscopy may be more time consuming than the examination itself. For the patient, "drop in" gastroscopy may reduce uncertainty, inadequate therapy and time off work.

**Methods:**

After an 8-9 month run-in period we asked patients, hospital staff and GPs to fill in a questionnaire to evaluate their experience with "drop in" gastroscopy and gastroscopy by appointment, respectively. The diagnostic gain was evaluated.

**Results:**

112 patients had "drop in" gastroscopy and 101 gastroscopy by appointment. The number of "drop in" patients varied between 3 and 12 per day (mean 6.5). Mean time from first GP consultation to gastroscopy was 3.6 weeks in the "drop in" group and 14 weeks in the appointment group. The half-yearly number of outpatient gastroscopies increased from 696 before introducing "drop in" to 1022 after (47% increase) and the proportion of examinations with pathological findings increased from 42% to 58%. Patients and GPs expressed great satisfaction with "drop in". Hospital staff also acclaimed although it caused more unpredictable working days with no additional staff.

**Conclusions:**

"Drop in" gastroscopy was introduced without increase in staff. The observed increase in gastroscopies was paralleled by a similar increase in pathological findings without any apparent disadvantages for other groups of patients. This should legitimise "drop in" outpatient gastroscopies, but it requires meticulous observation of possible unwanted effects when implemented.

## Background

A specialist health care reform was implemented in Norway in 2002 to facilitate referrals particularly for high-priority patients and reduce waiting time for specialist services. This aim was not met as the gain seemed to be limited to low rather than high priority patients [1]. By and large, waiting lists for gastrointestinal endoscopy are unduly long and with great variation between hospitals - 2 - 88 weeks for gastroscopy and 2-56 weeks for colonoscopy in the South-East Regional Health board area of Norway (Helse Sør-Øst) http://www.frittsykehusvalg.no-08/25/2010. Assessing priority of urgency for referred patients based on referral letters may be difficult and time consuming. Although the intentions may be good, the current prioritization practice has resulted in a skewed distribution in which women and the elderly are overrepresented among those who have been categorized as "low priority" [2]. Thus, it is pertinent to question the current allocation of resources into improvement of a failing priority policy.

There is controversy in the literature about the importance of so called "open access" gastroscopy, i.e. acceptance of referrals without spending time on further assessment of the indications given to have the procedure done [3-7]. Two studies claim a better outcome for patients with easier access to gastroscopy in case of gastric cancer [3] and peptic ulcer disease [4]. At the same time Sola-Vera et al [8] showed that patients' tendency to drop an appointment increases with the length of waiting time, which again might have impact on the patient's outcome.

A possible approach to the problem is to open up for a "free-access", "drop-in" endoscopy service [9]. On this background we decided to perform a pilot study of patient-controlled "drop in" gastroscopy to investigate the feasibility of removing all the priorities and see how the selection and findings would differ from the rest of the group of patients who were considered unsuitable for "drop in".

The aim of the study is to show that "drop in" outpatient clinic is feasible and that it may, for many of our patients, have the potential to shorten the waiting time to gastroscopy substantially, which would be a major improvement of our service.

## Methods

"Drop in" gastroscopy was defined as gastroscopy at the convenience of the patient with no appointment. The patient was required to appear fasting in defined sessions for "drop-in" (Monday - Thursday 0830-1000) with a referral letter from the patient's GP describing the clinical problem. This letter had to be no older than two weeks.

The present trial started 1. September 2008. It was announced among GPs in the hospital catchment area through "PK-news" (an online communication channel from practice consultants to GPs), personal e-mail and by letter. The doctors were given information about which groups of patients were not suitable for "drop in" gastroscopy. These patients were instead given an appointment as usual after assessing priority (patients with insulin dependent diabetes, warfarin treatment, infectious diseases (HIV, viral hepatitis, tuberculosis, heart valve failure or prosthesis with a need for antibiotic prophylaxis in connection with gastroscopy, need for general anesthesia or immigrants requiring translation services). Patients from the most remote parts of Telemark with a long journey to the hospital were also excluded from the scheme because of established hospital practice to coordinate logistics and transport for these patients to save costs.

During the inclusion period from 18.05.2009 to 26.06.2009, ie 8-9 months after having started the trial scheme, all "drop in" patients and all patients with appointments for gastroscopy were asked to fill in a questionnaire (additional file 1: questionnaire 1A and 1B respectively) on general satisfaction with the treatment and information given, which all of the patients did right after the endoscopic procedure. We focused specifically on the patient's acceptance of the time between attendance and gastroscopy in the "drop in" group and the time waiting for an appointment in the "appointment-group". All patients were asked about the time from the first symptom to the first visit to the GP and then to gastroscopy, and whether they had symptomatic treatment with "ulcer medicine" prescribed in the waiting period (Proton pump inhibitor (PPI), antihistamins (H2 inhibitors)).

During June 2009, all referring GPs, clinicians and nurses received a questionnaire (additional file 1: questionnaire 2 and 3 repectively) asking whether they were satisfied with the organization of outpatient gastroscopy. We also asked GPs if they wanted a purely technical endoscopic procedure or a more complete gastroenterological consultation in connection with the gastroscopy.

Questionnaire repliers were anonymous, but gender was specified and age was registered in three age categories.

### Statistics

Patients in the "Drop in" group and in the appointment group were selected based on certain criteria. Nevertheless we wanted to use statistical methods for visualization of expected differences - Chi-square for categorical variables and Student's t- test for assumed continuous variables.

Statistical processing was carried out with SPSS 15.0 (Chicago, Illinois, USA).

The project was approved by the Norwegian Social Science Data Services (NSD - 21,415). Being a quality assurance project on patient logistics it was not subject to assessment by the regional ethics committee for medical research.

## Results

112 patients were examined by "drop in" and 101 by appointment. Number of "drop in" gastroscopies per day varied between 3 and 12 (average 6.5). Age and gender distribution were comparable and there was no statistically significant difference between groups in the use of PPI and H2-inhibitors before gastroscopy (Table 1).

**Table 1:** Patient Characteristics

	"Drop in" gastroscopy	Gastroscopy by appointment	p-values
N	112	101	

Female (%)	66 (59%)	45 (45%)	P = 0.06

Age (years):			
18-35 (%)	21 (19%)	14 (14%)	P = 0.10
36-55	41 (37%)	28 (28%)	
> 55	44 (39%)	55 (55%)	
not replied	6 (5.4%)	4 (4.0%)	

Use of PPIs or H2 inhibitors prior to gastroscopy			
Yes (%)	36 (32%)	38 (37,6%)	P = 0.64
No	71 (63%)	57 (56,4%)	
Don't know	4 (3.6%)	4 (4.0%)	
Not replied	1 (0.9%)	2 (2.0%)	

Mean time from 1. symptom to 1. consultation of the GP (months/SD)	7.9/18.2	9.5/1.3	P = 0.97

Mean time from 1. consultation of the GP to Gastroscopy (weeks/SD)	3.6/9.7	13.9/23.2	P = 0.002

Time from the first symptom to the first medical visit at the GP's was on average 8.5 (SD 18) months for all patients - 7.9 months (SD 18.2) in the "drop in" group and 9.5 months (SD 18.3) in the appointment group (p = 0.63). Average time from first contact with the GP to gastroscopy was 3.6 weeks (SD 9.7) in the "drop in" group and 14 weeks (SD 23.2) in the appointment group (p = 0.002) (Table 1).

No adverse events due to gastroscopy activities were registered during the inclusion period.

In the "drop in" group 18% felt that the waiting time on the day of attendance was too long. The individual waiting times were not registered, but according to the length of the "drop-in" endoscopy sessions it could be no more than 4 hours. 15% in the appointment group felt that the time from referral to appointment was too long. 92% in the "drop in" group and 80% in the appointment group would prefer the same offer if requiring gastroscopy again at a later date. In both groups, more than 90% of patients were satisfied with the treatment (99-100%) and the information they received at gastroscopy (92-94%) (Table 2).

**Table 2:** Patient's satisfaction with the provided service

	Time to appointment	Time to wait when attended	Wishes for a possible later gastroscopy	Satisfied with Treat-ment Innforma-tion	
"Drop in" group		To long 17.9% Not to long 77.7% Don't know 4.4%	Appointment 8.3% "Drop in" 91.7%	99.1%	92%

Appointment-group	To long 15% Adequate 79% Don't know 6%		Appointment 80% "Drop in" 20%	100%	93.9%

59 (69%) out of the 85 referring GPs responded with questionnaire replies. 58 (98%) of these were satisfied with the "drop in" gastroscopy and the reports after gastroscopy. 27 (46%) wanted to retain control of the patient's examination programme and considered the gastroscopy to be a purely technical service. 27 (46%) wished the endocopist could take over the primary responsibility for the clinical assessment of the patient. Five (8.6%) wanted both, depending on the issue. In further comments 87% of GPs acclaimed the easy access and quick service of "drop in". Other comments given were: less administration (8.8%), fewer sick leaves (3.4%), fewer symptomatic treatments (1.7%) and ease of motivating patients to have an examination while symptoms were present (1.7%). Two, however, were concerned with overuse of gastroscopy and long waiting time on the day of attendance (3.4%).

10 of 11 employees at the gastroscopy unit (doctors and nurses) completed the questionnaire. Of these, 8 were satisfied with the re-organized service. Nine believed it was appropriate to maintain the "drop-in" offer although both doctors and nurses complained about the unpredictable workload.

After the "drop in" clinic was started in september 2008 the total number of outpatient gastroscopies increased to 1022 in January - June 2009 compared to 696 in the same months of 2008 (47% increase). The number of non-outpatient gastroscopies remained unchanged (452 vs. 450). However, there was also a corresponding increase in the number of pathological findings (Table 3). In particular, the number of patients diagnosed with reflux esophagitis and ulcer disease increased (Table 3). The total number of gastroenterological examinations at our outpatient clinic (gastroscopy and colonoscopy) increased from 1909 in the first six months of 2008 to 2284 in the first half of 2009. This means that the number of colonoscopies also increased from 761 to 810.

**Table 3:** Number of diagnoses (% of total number of gastroscopies in bracketts) in outpatient gastroscopies the first half of 2008 (before) and 2009 (after starting the "drop in" project)

	Total number of outpatient gastroscopies		p-values
	**1.1. -30.6.2008** **696**	**1.1.-30.6.2009** **1022**	

Gastric ulcer	26(3.7)	58(5.7)	0.069

Duodenal ulcer	17(2.4)	38(3.7)	0.14

Esophageal cancer	5(0.7)	9(0.9)	0.713

Gastric cancer	8(1.2)	21(2.1)	0.153

Esophagitis	217(31)	443(43)	< 0.001

Barrett's esophagus	17(2.5)	25(2.4)	0.996

Total	290(42)	594(58)	0.49

The trial resulted in no increase in personal resources in the endoscopy unit. In 2009 5600 procedures including 2716 gastroscopies, 1481 colonoscopies and 148 ERCPs were performed by a staff of 5 physicians, 4.2 nurses and 1 secretary.

## Discussion

"Drop in" gastroscopy is feasible within available resource limits. The staff at the endoscopy unit was willing to extend the testing period for drop in gastroscopy even if it meant more unpredictable workloads and increased volume with no increase in personnel. Advantages were: a better service to patients (short waiting time), reduced time spent on assessment and prioritization of referral letters as well as an increased volume of procedures which were matched by an increase in pathological findings of clinical significance (Table 3). Further, the re-organization of the service also reduced the problem of non-appearance for appointments. According to our previous experience up to 5% of patients do not attend for their gastroscopy appointment. This problem has been shown to be more serious with increasing waiting time from referral to time of appointment [8].

The main advantage, however, is an improved service to the patient which is a declared aim proved difficult to reach for health care providers.

However, the patients' questionnaire was not able to give detailed and specified estimates of patient satisfaction as it was not designed to do so. Nevertheless, the questionnaire results clearly suggest that organizing outpatient gastroscopy as a "drop in" service is at least comparable to gastroscopy by appointment in terms of patients' acceptance.

A major concern at the start of our project was the fear of having to face an unmanageable number of "drop in" patients each morning, even though the introduction of a similar scheme in Örebro in Sweden did not lead to an increased gastroscopy-volume [9]. However, the workload at an endoscopic unit is unpredictable anyway with a number of emergency patients to be fitted into a daily program that is already tight because of a national waiting list problem that also has affected Telemark county. "Drop in" patients can be regarded as a part of the same, normal unpredictability. As our staff got used to the new scheme, the burden of increased unpredictability became less important - and even the "unpredictability" of number of "drop in" patients became more predictable with time as attendance patterns emerged specific for each day of the week.

The increased number of procedures is justified by a simultaneous disproportionate increase in number of diagnoses, especially esophagitis and to some extent peptic ulcers. There might be claimed a risk of bias by endoscopists wishing to find lesions in the "drop in" group, at least for esophagitis. We were aware of this problem during our endoscopic procedures.

There are two clear concerns about the "drop in". The first is that patients, who are not suitable for "drop in" gastroscopy, but have a more urgent need for a gastroscopy, may be given lower priority. Even 8-9 months after starting the project, the average waiting time for a gastroscopy by appointment is still five weeks at the Hospital of Telemark (2-88 weeks in the whole area of South-East Norway) although the number of patients on the list decreased substantially. In this context it might be of importance that comorbidity in elderly patients can lead to an overrepresentation in the appointment group. In our study there was no skewed distribution of age and gender composition, but a larger patient sample might have demonstrated a difference. On the other hand, there was a non-significant overrepresentation of women in the "drop in" group, which might relate to earlier findings of women being overrepresented in the "low priority" group [2]. If "drop-in" can contribute to easier access for low-priority groups with high medical needs, this would be in line with a policy of equality for health care services.

The other concern is that the increased number of gastroscopies due to increased referral to our center may be at the expense of other procedures such as colonoscopies. This was not the case in our unit as the number of colonoscopies also increased during the period.

We had expected a lower consumption of PPIs and H2 inhibitors among the "drop in" patients because of reduced time from GP-consultation to gastroscopy. The average time from first contact to the GP for the current problems of gastroscopy (3.6 weeks) was longer than the two weeks deadline between the date of the GP referral letter and appearance for "drop in", because the GPs did not refer an endoscopy by the first contact with the patient. In fact, the large SD of 9.7 indicates a substantial individual degree of "doctor's delay". This may indicate that a "wait-and-see" policy with prescription of acid secretion inhibitors may be difficult to change even after 8-9 months' break-in period for the "drop in" service. There is an economic potential in the ability to avoid inappropriate, symptomatic use of acid-inhibiting drugs by early diagnostic accuracy through a "drop in" service, which has not been utilized yet. The significantly shorter time from first medical contact to gastroscopy in the "drop in" group can also be thought to influence the course of the disease (prognosis and sick leave periods).

Some studies suggest that survival in gastric cancer [5] and mortality due to NSAIDs-related peptic ulcer [6] can be improved in connection with the organizational measures to reduce waiting times for gastroscopy. Other studies have not been able to confirm this effect and our pilot study was not designed to show this [7-9]. Table 3 shows more diagnosed gastric cancers in 2009 compared to 2008. We have no explanation for the phenomenon which may be a chance finding.

## Conclusion

"Drop in" outpatient clinic for gastroscopy is a satisfactory form of organization that saves resources, especially for management of patient referrals and waiting lists. Patients, GPs and staff at our endoscopy unit were satisfied with the arrangement.

In connection with the increased number of procedures, the number of relevant diagnoses increased as well. This is in itself a significant argument for implementation of "drop in" gastroscopy as a permanent service, but it requires monitoring of possible undesirable effects.

## Competing interests

The authors declare that they have no competing interests.

## Authors' contributions

GHH is responsible for the study design, drafted the manuscript, participated in the data collection, performed statistic analysis

LC, SD, AJ, VM, JP participated in the data collection, revised the manuscript.

GH participated in the study design and drafting of the manuscript, participated in statistic analysis.

All authors have read and approved the final manuscript

## Pre-publication history

The pre-publication history for this paper can be accessed here:

http://www.biomedcentral.com/1471-230X/12/12/prepub

## Supplementary Material

Additional file 1 **Questionnaires**. 1. Questionnaire (1 A) for patients after gastroscopy ("drop in" group) version 25.02.09 2. Questionnaire (1 B) for patients after gastroscopy (appointment group) 3. Questionnaire (2) for GPs who refer to outpatient clinic for gastroscopy. 4. Evaluation of "drop in gastroscopy" - questionnaire for the staff at the clinic (3). Click here for file
