# Supplementary material for: "Drop in" gastroscopy outpatient clinic - experience after 9 months
Source: BMC Gastroenterol. 2012 Feb 1;12:12. doi: 10.1186/1471-230X-12-12 (PMC3293713; doi:10.1186/1471-230X-12-12)
Supplement: Additional file 1 — Questionnaires. 1. Questionnaire (1 A) for patients after gastroscopy ("drop in" group) version 25.02.09 2. Questionnaire (1 B) for patients after gastroscopy (appointment group) 3. Questionnaire (2) for GPs who refer to outpatient clinic for gastroscopy. 4. Evaluation of "drop in gastroscopy" - questionnaire for the staff at the clinic (3). [file 1471-230X-12-12-S1.DOC]

**Additional file - Questionnaires**

**Questionnaire (1 A) for patients after gastroscopy (“drop in” group)
version 25.02.09**

1. Are you satisfied with the treatment you received today?

⁪ Yes ⁪ No ⁪ Do not know

2. If not - what is the reason?

 .................................................................................................................................

3. Was the information you received today in connection with the survey satisfactory?

⁪ Yes ⁪ No ⁪ Do not know

4. Are you satisfied with the information folder you have got from your GP about “drop in” gastroscopy?

⁪ Yes ⁪ No ⁪ Do not know

5. Do you think the time from attending at the unit today until the gastroscopy was too long?

Yes ⁪ ⁪ No ⁪ Do not know

6. If you could choose in case of a new gastroscopy, would you prefer:

 an appointment even if that means several weeks of waiting?

a "drop in" gastroscopy at a day you decide (like the one you had now), even if you

risk having to sit and wait 2

hours before it is your turn?

7. How much time has passed from the time you felt the first symptoms until you went to the

GP ? ..........

8. How much time has passed from your first visit to the doctor until the gastroscopy today?

.................

9. Did you use some kind of "ulcer-drug" during that time?

⁪ Yes ⁪ No ⁪ Do not know

10. If you remember: what kind of "ulcer medicine" did you use?

11. Your age: ⁯ 18-35 ⁯ 36-55 ⁯ more than 55

12. You are: ⁯ Male ⁯ Female

**Questionnaire (1 B) for patients after gastroscopy (appointment group)
version 25.02.09**


1. Are you satisfied with the treatment you received today?

⁪ Yes ⁪ No ⁪ Do not know

2. If not - what is the reason?


3. Was the information you received today in connection with the survey satisfactory?

⁪ Yes ⁪ No ⁪ Do not know

4. What do you think about the time from referral to the appointment for gastroscopy?
Too long ⁪ ⁪ Adequate ⁪ Do not know

5. If you could choose in case of a new gastroscopy, would you prefer (please, tick off):

 an appointment like the one you had now, even if that means several weeks of waiting?

a "drop in" gastroscopy at a day you decide, even if you risk having to sit and wait 2 hours before it is your turn?

6. How much time has passed from the time you felt the first symptoms until you went to the GP ?..........

7. How much time has passed from your first visit to the doctor until the gastroscopy today? .................

8. Did you use some kind of "ulcer-drug" during that time?

⁪ Yes ⁪ No ⁪ Do not know

9. If you remember: what kind of "ulcer medicine" did you use?


10. Your age: ⁯ 18-35 ⁯ 36-55 ⁯ more than 55

11. You are: ⁯ Male ⁯ Female

**Questionnaire (2) for GPs who refer to outpatient clinic for gastroscopy.
version 25.02.09**
1. What is your overall assessment of "drop in gastroscopy" - are you satisfied?

⁪ Yes ⁪ No ⁪ Do not know

2. If not, what is the reason?

 ............................................................................................................................

3. Are you satisfied with the service that we provide when patients show up?

⁪ Yes ⁪ No ⁪ Do not know

4. If not, what is the reason?

............................................................................................................................

5. Are you satisfied with our reports after "drop in gastroscopy"?

⁪ Yes ⁪ No ⁪ Do not know

6. If not, what can we do better?

.................................................................................................................................

7. We discuss how we can meet your needs as a family doctor in the best possible way. What

would you prefer (please, tick off)?

⁪ We perform the gastroscopy, give possibly some advice regarding any further
     assessment and treatment, leaving, however, the responsibility for further follow-up to

you.

⁪ We perform the gastroscopy, and – if necessary - take over the responsibility for further

assessment of the patient’s health problem.

⁯ Other? (please, specify)... ... ... ... ... ... ... ... ... ... ... ... ... ... ... ... ... ... ... ... ... ... ... ... ... ... .

8. What advantages do you see with "drop in" service? Some key words:

 .........................................................................................................................................

9. What disadvantages do you see with "drop in" service? Some key words:

 .........................................................................................................................................

**Evaluation of "drop in gastroscopy" - questionnaire for the staff at the clinic (3)
version 25.02.09**
1. What is your overall assessment of "drop in gastroscopy" - are you satisfied ?

⁪ Yes ⁪ No ⁪ Do not know

2. If not - what is the reason?

... ... ... ... ... ... ... ... ... ... ... ... ... ... ... ... ... ... ... ... ... ... ... ... ... ... ... ... ... ... ... ... ....

3. Do you think that “drop in” gastroscopy is a valuable service for patients?

⁪ Yes ⁪ No ⁪ Do not know

4. Do you think that “drop in” gastroscopy is a good service for GPs?

⁪ Yes ⁪ No ⁪ Do not know

5. Should we continue with this kind of service?

⁪ Yes ⁪ No ⁪ Do not know

6. If not - why not?

 ..........................................................................................................................................

7. Do you have any suggestions for improvement?

…………………………………………………………………………………………………..


8. What are the advantages of the service?

... ... ... ... ... ... ... ... ... ... ... ... ... ... ... ... ... ... ... ... ... ... ... ... ... ... ... ... ... ... ... ... ...

9. What are the disadvantages of the service?

... ... ... ... ... ... ... ... ... ... ... ... ... ... ... ... ... ... ... ... ... ... ... ... ... ... ... ... ... ... ... ... ....
